# Estimation of marginal structural models under irregular visits and unmeasured confounder: calibrated inverse probability weights

**DOI:** 10.1186/s12874-022-01831-2

**Published:** 2023-01-07

**Authors:** Sumeet Kalia, Olli Saarela, Michael Escobar, Rahim Moineddin, Michelle Greiver

**Affiliations:** 1 Dalla Lana School of Public Health, University of Toronto, Toronto, Canada; 2 Department of family and community medicine, University of Toronto, Toronto, Canada

**Keywords:** Longitudinal data analysis, Marginal structural models, Calibrated weights, Constrained optimization, Electronic health records, Primary care

## Abstract

Clinical information collected in electronic health records (EHRs) is becoming an essential source to emulate randomized experiments. Since patients do not interact with the healthcare system at random, the longitudinal information in large observational databases must account for irregular visits. Moreover, we need to also account for subject-specific unmeasured confounders which may act as a common cause for treatment assignment mechanism (e.g. glucose-lowering medications) while also influencing the outcome (e.g. Hemoglobin A1c). We used the calibration of longitudinal weights to improve the finite sample properties and to account for subject-specific unmeasured confounders. A Monte Carlo simulation study is conducted to evaluate the performance of calibrated inverse probability estimators using time-dependent treatment assignment and irregular visits with subject-specific unmeasured confounders. The simulation study showed that the longitudinal weights with calibrated restrictions improved the finite sample bias when compared to the stabilized weights. The application of the calibrated weights is demonstrated using the exposure of glucose lowering medications and the longitudinal outcome of Hemoglobin A1c. Our results support the effectiveness of glucose lowering medications in reducing Hemoglobin A1c among type II diabetes patients with elevated glycemic index ($\ge 8.5 \%$) using stabilized and calibrated weights.

**Supplementary Information:**

The online version contains supplementary material available at 10.1186/s12874-022-01831-2.

## Introduction

Prospective randomized experiments are conducted to evaluate the effectiveness of an intervention. However, in the absence of controlled experiments, large data repositories may provide an alternative source to emulate the randomized experiment with the intention to draw causal inference pertaining to the effectiveness of an intervention [1]. Longitudinal data can be collected at fixed (or pre-specified) time points or irregular time points during the study follow-up. For example, a randomized controlled study may pre-specify the fixed follow-up visits in advance at regular time points, while the data collection at unequally spaced visit times in an observational data repository may be related to the outcome. Since patients do not interact with the health care system at random, the longitudinal information collected in electronic health records (EHRs) tend to exhibit bias in the form of informative visit regimen [2]. For example, the data collected in EHRs may correspond to an over-representation of patient population with severe or worse disease symptoms when patients are only visiting the clinic with the acute onset of new medical ailment or the management of pre-existing chronic condition. The outcomes and the visits in discrete time-intervals may be correlated in which case the application of standard regression model (e.g. generalized estimating equations) generates biased estimation [3, 4]. To correct for this, Pullenayegum and Lim [5] outlined two methods to handle longitudinal data with irregular visits: (i) inverse-intensity weighting and (ii) model-based approach using correlated random effects between the visits and the outcome. Inverse-intensity weighting method uses the measured covariates to capture the correlation between the visit and outcome processes. Similarly, the semi-parametric method assumes the dependency between the visits and outcome through baseline covariates and time-varying latent factors.

Pullenayegum and Lim [5] describe four categories of visit process: (i) fixed visits, (ii) history-dependent protocol visit, (iii) physician-driven visit, (iv) patient-driven visit. We limit the focus of this article to “history-dependent protocol visit” in which the protocol specify when the visits should be, but the intervals between visits are allowed to depend on patients’ previously observed history [5]. As an example, we may specify the covariate-dependent irregular visits are more frequent in the presence of confounding factors [5]. In our context, history-dependent protocol visit is a reasonable assumption for the management of patients with diabetes, since the patients are expected to visit the primary care clinic routinely depending on their health status, and as recommended by Diabetes Canada guidelines [6]. We describe the irregular visits as the presence of missed visits that do not take place within an interval (i.e. missed visits). We use non-overlapping intervals to discretise the continuous-time processes, and we conceptualize treatment, confounder and outcome as random variables that evolve with study follow-up. We assume that the history-dependent protocol visit characterize the missing at random mechanism in which the missing outcome is not dependent on current and future outcome conditional on the past outcome and covariates [5]. We may quantify the extent of visit irregularity by examining the deviation from perfect repeat-measures (i.e. one visit per time interval) using the proportion of missed visits within each time intervals [7].

In the context of longitudinal causal inference, Hernán et al. [8] considered subject-specific visit times using a static observation plan with pre-specified regular times and dynamic observation plan with irregular visit occurrence due to clinical evolution of patients. Hernán et al. [8] demonstrated the need to account for selection bias and confounding arising in estimation of the effects of a dynamic treatment regime. The dependence between the outcome and the visit can arise under various combinations including: (i) conditional independence given past outcome-model covariates, (ii) conditional independence given past observation-time model covariates, (iii) conditional independence given shared latent variables [9]. In this article, the dependence between the treatment and the outcome is induced by an unmeasured (subject-specific) confounder, and thereby treatment is assumed to influence the outcome after conditioning on the observed history [10].

Regression models may yield biased estimation of longitudinal causal effect of time-varying treatment in the presence of time-varying confounder and treatment-confounder feedback [11]. In particular, selection bias is introduced when standard methods (e.g. generalized estimating equations) are used to adjust for the effect of time-dependent confounding. The existence of treatment-confounder feedback (i.e. past treatment affects the current confounder which affects the current treatment) requires the use of causal inference methods to estimate the causal effect of time-dependent treatment [12]. In addition to treatment-confounder feedback, the longitudinal data collected in EHRs may also contain additional feedback with respect to visit-confounder where the past visit affects the current confounder which affects the future visit. Marginal structural models using inverse-probability weights with respect to treatment and visit account for such temporal feedback, and provide consistent estimates of marginal causal effects with correct model specification and without unmeasured confounding factors.

### Knowledge gap and motivating example

To our knowledge, the simultaneous existence of treatment-confounder and visit-confounder feedback with unmeasured confounder for each subject in longitudinal settings has not been considered in the causal inference literature. The cumulative-time weights with respect to treatment and visit account for this feedback. Moreover, the treatment weights and visit weights are calibrated to make the non-probability (i.e. observational) sample similar to the target population. In this article, we extend the earlier work by Yiu and Su [13]) in two-folds: (i) extending calibration of longitudinal weights to irregular visits, (ii) incorporating unmeasured confounder for each subject in longitudinal design. The application of this method is demonstrated to assess the effectiveness of glucose lowering medications among diabetes patients with elevated glycemic index (Hemoglobin A1c $\ge 8.5 \%)$.

## Methods

### Notation

A framework for longitudinal causal inference is considered for *n* individuals $(i=1,...,n)$ in $m_i$ time-intervals $(j=1,...,m_i)$. An equally spaced time interval is denoted as $\{T_{i1}< ... < T_{ik_i}\}$. The time-dependent binary treatment is denoted as $A_i(T_{ij})=A_{ij}$, time-dependent binary confounder is denoted as $L_i(T_{ij})=L_{ij}$, the occurrence of visit is denoted as $V_i(T_{ij})=V_{ij}$, the continuous outcome is denoted as $Y_i(T_{ij})=Y_{ij}$. The vector of baseline covariates for individual *i* is denoted as $X_{i}$. The latent confounder $\eta _i$ is defined as a common cause of treatment $A_{ij}$ and outcome $Y_{ij}$ for individual *i*. In some instances, the index for individual *i* is suppressed because it is assumed that the random vector for each individual *i* is drawn independently with respect to other individuals. The cumulative treatment status $\bar{A}_{ij}$, cumulative confounder status $\bar{L}_{ij}$, cumulative visit status $\bar{V}_{ij}$ denotes the complete history of each factor up to and including visit *j*. As an example, $\bar{A}_{ij}= \{A_{i0},A_{i1},...A_{ij}\}$; $\bar{L}_{ij}= \{L_{i0},L_{i1},...L_{ij}\}$; $\bar{V}_{ij}= \{V_{i0},V_{i1},...V_{ij}\}$. We denote observed history as $\bar{{H}}_{j-1} \equiv \{\bar{V}_{j-1}, \bar{Y}_{j-1}, \bar{L}_{j-1},\bar{A}_{j-1},X\}$. We introduce $Y^{\bar{a},\bar{v}}_j$ and $L_j^{\bar{a},\bar{v}}$ as potential outcome and potential confounder (respectively) under treatment regime $\bar{a}$ and visit regime $\bar{v}$ with respect to the $j^{th}$ visit.

### Marginal structural model

The marginal causal effects of treatment are specified through a parametric marginal structural model with respect to the longitudinal outcome as

1 $$\begin{aligned} g^{-1}(E( Y_{ij}^{\bar{a},\bar{v}} | X_i )) = h(\bar{a},\bar{v}, X_i; \theta )) \end{aligned}$$

where $Y_j^{\bar{a},\bar{v}}$ is the potential outcome indexed with respect to hypothetical treatment $\bar{a}$ and hypothetical visit $\bar{v}$. The link function is represented using an identity function $g^{-1}(\cdot )$. We assume a rank-preserving model in which the net change in potential outcome (i.e. $E( Y_j^{\bar{a},\bar{v}} | \bar{{H}}^*_{j-1})$) is preserved with respect to the treatment and visit for all subjects (i.e. absence of effect modification) [14]. In the context of EHRs, the causal contrast correspond to net change in HbA1c with respect to glucose-lowering medications. We are interested in accounting for three sources biases that distort the causal estimator: (i) measured confounding arising due to time-dependent covariates, (ii) selection bias arising due to irregular visits (i.e. missing at random), and (iii) subject-specific unmeasured confounder.

We use the generalized estimating equations with inverse probability weights to obtain an estimate of the marginal treatment effect with respect to time-dependent covariates. We describe the marginal structural model using the score function of weighted generalized estimating equations as

2 $$\begin{aligned} S(\beta )= \sum\limits_{i=1}^n \frac{\partial \mu _i^\prime }{\partial \beta } \nu _i^{-1} SW_i (Y_i - \mu _i(\beta )) = 0 \end{aligned}$$

where $SW_i$ are the inverse probability weights with stabilizing factor, $\nu _i$ is the working covariance matrix of $Y_i$ and it is specified through working correlation matrix $R(\alpha )$, and $\mu _i(\beta )$ is the mean vector. The correct specification of the correlation matrix $R(\alpha )$ improves the efficiency of estimation while misspecification may still lead to consistent estimators.

The longitudinal weights with stabilization factor are constructed to account for treatment-confounder and visit-confounder feedback as

$$\begin{aligned} SW^{\bar{A}}_t=\prod\limits_{j=1}^{t} \frac{ Pr(A_{j}|\bar{{H}}^*_{j-1} )}{ Pr(A_{j}|\bar{H}_{j-1} )}; \quad SW^{\bar{V}}_t=\prod\limits_{j=1}^{t} \frac{ Pr(V_{j}| \bar{{H}}^{**}_{j-1})}{ Pr(V_{j}| \bar{H}_{j-1})} \end{aligned}$$

$$\begin{aligned} SW^{\bar{A},\bar{V}}_t= SW_t^{\bar{A}} \times SW_t^{\bar{V}}. \end{aligned}$$

where the product terms are defined over cumulative discrete-time intervals. The observed partial history with the exclusion of time-dependent covariates for treatment and visits is denoted as $$\bar{{H}}^*_{j-1}$$ and $$\bar{{H}}^{**}_{j-1}$$. Since the time-varying treatment $$A_j$$ and time-varying visit $$V_j$$ are statistically endogenous, the stabilized inverse probability treatment weights $$SW^{\bar{A}}_t$$, stabilized inverse probability visit weights $$SW^{\bar{V}}_t$$ and joint inverse probability weights $$SW^{\bar{A},\bar{V}}_t$$ are required to estimate the marginal causal effect of glucose-lowering medications on hemoglobin A1c. The stabilized treatment weights are used to create a pseudo-population in which the imbalance for time-dependent covariates across treatment groups is reduced. The stabilized visit weights are used to create a pseudo-population in which the selection bias due to irregular visits (missing at random) is reduced. The pseudo-population generated using the joint weights $$SW^{\bar{A},\bar{V}}_t$$ incorporate both sources of biases (i.e. confounding bias and selection bias).

### Assumptions

Longitudinal causal inference with time-varying treatment use the sequential version of identifiability assumptions: (i) latent sequential exchangeability, (ii) sequential postivity and (iii) sequential consistency. The sequential exchangeability assumption is an extension of conditional exchangeability (or no unmeasured confounding) assumption where the probability of treatment assignment and visit occurrence at $j^{th}$ visit depends on past treatment, past visit and covariate history, and conditional on these factors the potential outcome and potential confounder is independent of the treatment assignment. The latent sequential exchangeability assumption (or equivalently latent ignorability assumption) can be described as

$$\begin{aligned} \{Y^{\bar{a},\bar{v}}_{j}, L^{\bar{a},\bar{v}}_{j} \}{\perp \! \! \! \perp } \{A_{j}, V_{j} \}| \{ \bar{{H}}_{j-1}, \eta _{i}\}. \end{aligned}$$

The sequential positivity assumption is defined as a non-zero probability of treatment assignment and observed visit at each time interval *j* given the history $\bar{{H}}_{j-1}$. We describe sequential positivity as $P(A_j |\bar{{H}}_{j-1}) >0$ and $P(V_j| \bar{{H}}_{j-1}) > 0$ for $\forall \bar{a}, \bar{v}$. The sequential consistency assumption links the potential outcome $Y_j^{\bar{a},\bar{v}}$ and confounder $L_j^{\bar{a},\bar{v}}$ to the observed outcome and confounder as

$$\begin{aligned} Y_{j}^{\bar{a},\bar{v}}=Y_{j} \quad \quad L_{j}^{\bar{a},\bar{v}}=L_j \quad \quad \text {if}\ \bar{A}=\bar{a}\ \text {and}\ \bar{V}=\bar{v}. \end{aligned}$$

If causal identifiability assumptions are satisfied then an observational study can be used to mimic a randomized experiment with the limitation that the conditional probability of treatment assignment is unknown and need to be estimated using the data in an observational study. It is further assumed that the administrative censoring $C_{ij}$ is non-informative where censoring is independent of observation times $T_{ij}$ and longitudinal outcome $Y_{ij}$ conditioned on history $\bar{{H}}_{j-1}$ as $C_{ij} {\perp \! \! \! \perp } \{Y_{ij},T_{ij}\} | \{ \bar{{H}}_{j-1}, \eta _{i}\}$.

### Calibration of inverse probability weights

We motivate the application of calibrated longitudinal weights to estimate the causal effects of glucose lowering medications on hemoglobin A1c. Calibration has been used in causal inference framework to estimate the average treatment effect when regularizing high-dimensional covariates with lasso penalty [15], to minimize the variance of calibrated weights [16], to improve the finite sample properties of maximum likelihood estimation [17], to account for unmeasured cluster-level confounders [18].

Under finite longitudinal sample, stabilized inverse probability weights may not remove the associations between time-dependent treatment $A_{ij}$ and time-dependent covariate $\bar{L}_{ij}$ conditional on past treatment regimen $\bar{A}_{ij-1}$. There may still exist chance imbalances and residual confounding of covariates in the pseudo-population (weighted using $SW_{ij}^A$ or $SW_{ij}^V$) and this may contribute to finite sample estimation errors [19]. The sample estimation errors may further become exacerbated when the treatment model or the visit model are misspecified. In this article, the longitudinal inverse probability treatment and visit weights are calibrated to account for (i) associations between treatment regimen and time-dependent covariates, (ii) associations between irregular visits and time-dependent covariates, (iii) unmeasured subject-specific (i.e. time-invariant) confounder. The purpose of calibrating the longitudinal weights is to improve the small sample properties of the longitudinal weights [17], while accounting for the unmeasured subject-specific confounder and irregular visits.

The calibration restrictions are employed on inverse probability weights to make the treatment assignment unassociated with the history of the time-varying covariates at each time interval in the pseudo-population. Yiu and Su [17] proposed the calibrated inverse probability weights to improve estimation errors under finite samples using maximum likelihood estimation. Similar to Yiu and Su [17], we calibrate the treatment and visit inverse probability weights using the maximization of weighted log-likelihood functions:

$$\begin{aligned} \prod\limits_{i=1}^n \prod\limits_{j=1}^{m_i} \left\{ \prod\limits_{k=1}^j P_{\alpha _w}(A_{ik} | \bar{H}_{ik-1}; \alpha _w)\right\} ^{SW_{ij}^{A}(\lambda )} \end{aligned}$$

$$\begin{aligned} \prod\limits_{i=1}^n \prod\limits_{j=1}^{m_i} \left\{ \prod\limits_{k=1}^j P_{\omega _w}(V_{ik} | \bar{H}_{ik-1}; \omega _w)\right\} ^{SW_{ij}^{V}(\lambda )} \end{aligned}$$

after weighting the sample as

$$\begin{aligned} SW_{ij}^A({\lambda } )= SW_{ij}^A c(\bar{L}_{ij},{\lambda } ) \end{aligned}$$

$$\begin{aligned} SW_{ij}^V({\lambda } )=SW_{ij}^V c(\bar{L}_{ij},{\lambda } ) \end{aligned}$$

where $c(\bar{L}_{ij},{\lambda } )$ is the calibration function and reduces to one when the vector of coefficients ${\lambda }=0$ (i.e. $c(\bar{L}_{ij},{\lambda =0})=1)$. We assume a non-negative parametric form of $c(\bar{L}_{ij},{\lambda } )= exp(K\lambda )$, where the matrix $K \in \mathbb {R}^{N \times r}$ includes the data-dependent restrictions with $N=\sum _{i=1}^n m_i$ observations and *r* columns. The unknown vector of $\lambda \in \mathbb {R}^{r}$ is estimated using the calibration of inverse probability treatment and visit weights.

In similar spirit to Yiu and Su [20], the regression coefficient $\alpha _w$ of treatment model are partitioned into $\{\alpha _b,\alpha _d\}$, where $\alpha _b$ characterize the baseline distribution (e.g. intercept term) and $\alpha _d$ characterize the dependence of treatment assignment and covariates. Likewise, the regression coefficients of irregular visits $\omega _w$ are partitioned into $\{\omega _b, \omega _d\}$ with the same convention. We maximize the weighted log-likelihood function with respect to $\lambda$ through a score equation

3 $$\begin{aligned} \sum\limits_{i=1}^n \sum\limits_{j=1}^{m_i} SW_{ij}^A ({\lambda }) \sum\limits_{ k=1}^j \left. \frac{\partial }{\partial \alpha _w} log(P_{\alpha _w}(A_{ik} |\bar{H}_{ik-1}; \alpha _\omega )) \right| _{\alpha _b=\hat{\alpha }, \alpha _{d}=0} = 0 \end{aligned}$$

where the innermost index *k* accumulates over *j* time intervals. The dependence of treatment $A_{ij}$ with the time-dependent covariates $\bar{L}_{ij}$ is represented as $\alpha _d$ and it is constrained to be zero while the vector of treatment coefficients (excluding time-dependent covariates) is represented as $\alpha _b$ and it is set to the maximum likelihood estimates $\hat{\alpha }$. In addition to calibrated treatment weights, the visit weights are calibrated to reduce the association between irregular visits and time-dependent covariates through a score equation

4 $$\begin{aligned} \sum\limits_{i=1}^n \sum\limits_{j=1}^{m_i} SW_{ij}^V ({\lambda }) \sum\limits_{ k=1}^j \left. \frac{\partial }{\partial \omega _w} log(P_{\omega _w}(V_{ik} | \bar{H}_{ik-1};\omega _w)) \right| _{\omega _b=\hat{\omega }, \omega _{d}=0} = 0 \end{aligned}$$

where the vector of regression coefficients $\omega _{d}=0$ denote the independence of time-dependent covariate $\bar{L}_{i,j-1}$ and irregular visits $V_{ij}$ (constrained to be zero) while $\omega _b=\hat{\omega }$ is set to maximum likelihood estimates $\hat{\omega }$.

In both score Eqs. (3) and (4), we notice that the calibrated weights (i.e. $SW_{ij}^A(\lambda )$ and $SW_{ij}^V(\lambda )$) are used to weight the likelihood of treatment model and visit model (respectively) for the $i^{th}$ patient up to and including the time interval *j*. The calibration restrictions are inverted to estimate the values of unknown coefficients $\lambda$. The calibration restrictions using $\{\alpha _d=0\}$ and $\{\omega _d=0\}$ ensures that the treatment assignment and irregular visits are statistically exogenous with respect to the time-dependent covariates. Since the covariate balancing restrictions reduce the dependence for treatment assignment and irregular visits with respect to the functional covariate history $\tilde{L}_{ij}$, we may represent the model-based restrictions (derived in Appendix Section) as

$$\begin{aligned} \sum\limits_{i=1}^n \sum\limits_{j=1}^{m_i} SW_{ij}^A ({\lambda }) \sum\limits_{ k=1}^j (A_{ik} - \hat{e}_{ik}^A) \tilde{L}_{ik-1} = 0 \end{aligned}$$

and

$$\begin{aligned} \sum\limits_{i=1}^n \sum\limits_{j=1}^{m_i} SW_{ij}^V ({\lambda }) \sum\limits_{ k=1}^j (V_{ik} - \hat{e}_{ik}^V) \tilde{L}_{ik-1} = 0 \end{aligned}$$

where the model-based propensity scores for treatment model $\hat{e}_{ik}^A$ and visit model $\hat{e}_{ik}^V$ are estimated as $\hat{e}_{ij}^A=P_{\alpha }(A_{ij}| \bar{H}_{j-1}^*)$ and $\hat{e}_{ij}^V=P_{\omega }(V_{ij}| \bar{H}_{j-1}^*)$. The model-based covariate balancing restrictions are accumulated with respect to longitudinal observations. The residuals for treatment (i.e. $A_{ij} - \hat{e}_{ij}^A$) and irregular visit (i.e. $V_{ij} - \hat{e}_{ij}^V$) are set to be orthogonal with respect to the functional history of time-dependent covariates $\tilde{L}_{ij}$ (including intercept term) in the pseudo-population defined using the calibrated weights.

#### Unity mean restrictions

The stabilized inverse probability weights used in the pseudo-likelihood function of marginal structural models tend to satisfy the property of unity mean at each time interval (i.e. $E(SW_{j}^A)=E(SW_{j}^V)=1 \ \forall j$) [21]. However, this property is not guaranteed to hold for calibrated inverse probability weights. Thus, in addition to the covariate balancing score constraints, we further impose the restrictions for average calibrated weights to be one at each time interval as

$$\begin{aligned} E( SW_{ij}^{A}(\varvec{\lambda })) = \frac{1}{n} \sum\limits_{i=1}^n SW_{ij}^{A}(\varvec{\lambda })=1 \quad \quad E( SW_{ij}^{V}(\varvec{\lambda })) = \frac{1}{n} \sum\limits_{i=1}^n SW_{ij}^{V}(\varvec{\lambda })=1. \end{aligned}$$

The average treatment weights and visit weights are constrained to be equal to one at each time interval to stabilize the longitudinal weights and to prevent trivial solutions of zero (or negative weights) during the calibration procedure.

#### Time-invariant latent restrictions

In the presence of subject-level unmeasured confounder $\eta _i$, the exposed and the unexposed subjects (with respect to treatment) are not conditionally exchangeable given $\bar{H}_{j-1}$ because non-causal association between $\bar{A}_{j}$ and $Y_{j}$ cannot be blocked by conditioning on measured history $\bar{H}_{j-1}$. We derive the balancing constraints for subject-specific unmeasured confounder in the context of repeated-measures longitudinal outcomes in Appendix Section. We obtain the empirical constraints to account for the unmeasured individual-level confounder $\eta _i$ using treatment $A_{ij}$ as

5 $$\begin{aligned} \left\{ \begin{array}{ll} \sum _{i=1}^n \sum _{j=1}^{m_i} SW_{ij}^A ({\lambda }) \sum _{ k=1}^j \left[ (A_{ik} - \hat{e}^A_{ik}) \times L_{ik-1} \right] &{}= 0\\ \sum _{j=1}^{m_i} SW_{ij}^A ({\lambda }) \sum _{ k=1}^j \left[ (A_{ik} - \hat{e}^A_{ik}) \right] &{}= 0 \quad \quad \forall i \end{array}\right. \end{aligned}$$

and using visit $V_{ij}$ as

6 $$\begin{aligned} \left\{ \begin{array}{ll} \sum _{i=1}^n \sum _{j=1}^{m_i} SW_{ij}^V ({\lambda }) \sum _{ k=1}^j\left[ (V_{ik} - \hat{e}^V_{ik}) \times L_{ik-1} \right] &{}= 0\\ \sum _{j=1}^{m_i} SW_{ij}^V ({\lambda }) \sum _{ k=1}^j \left[ (V_{ik} - \hat{e}^V_{ik}) \right] &{}= 0 \quad \quad \forall i \end{array}\right. \end{aligned}$$

The empirical constraints in Eqs. (5) and (6) are sufficient to describe the covariate balancing restrictions with respect to the time-invariant latent confounder $\eta _i$. These empirical restrictions balance the time-dependent covariate distribution across treatment groups and visit indication within each time interval in the presence of subject-specific latent confounder.

## Simulation study

Kang et al. [22] assessed the performance of various methods that use inverse probability weighting to estimate the causal effect from observational data with incomplete (missing) outcome. In similar spirit to Kang et al. [22] and others [16, 23, 24], the data generation in this simulation study is designed as an amalgamation of earlier longitudinal settings to evaluate the performance of inverse probability weighting with calibration restrictions.

### Estimation of marginal treatment effect

We allow the treatment $A_{j}$ to depend on the evolution of an individual’s time-varying covariate(s) $\bar{L}_{j-1}$, and also $\bar{A}_{j-1}$. Using the potential outcome framework defined with respect to treatment $\bar{A}_j$ and visit $\bar{V}_j$, the non-null causal effect is estimated using the G formula as

$$\begin{aligned} E(Y_{j}^{\bar{a},\bar{v}} |X=x) = \sum\limits_{\forall \bar{l}_{j-1}}\sum\limits_{\forall \bar{y}_{j-1} } E(Y_{j}|\bar{H}_{j-1} ) \prod\limits_{k=1}^{j-1} E(Y_k| \bar{H}_{k-1}) P(L_k=l_k | \bar{H}_{k-1}) \end{aligned}$$

where the sum is defined with respect to all possible realizations of $\{\bar{y}_{j-1}, \bar{l}_{j-1}\}$ and the marginal means of potential outcome $E(Y_{j}^{\bar{a},\bar{v}} |X=x)$ is specified with respect to treatment $\bar{A}_j=\bar{a}_j$ and visit $\bar{V}_j=\bar{v}_j$. With large number of time intervals, the marginal means of potential outcomes may also be approximated using Markov and stationary assumptions [25].

Apart from observed dependencies between outcome $E(Y_j |\bar{H}_{j-1})$ and treatment $E(A_j |\bar{H}_{j-1})$ using observed history $\bar{H}_{j-1}$, a subject-specific random effect $\eta _i$ generates latent dependency between the outcome $E(Y_j |\bar{H}_{j-1} ,\eta )$ and the time-dependent treatment $E(A_j|\bar{H}_{j-1} ,\eta )$. As detailed in Appendix section, the G-computation can be extended to incorporate the subject-specific random effect $\eta _i$ as

$$\begin{aligned} E(Y_{j}^{\bar{a},\bar{v}} |X_i) = \int \sum\limits_{\forall \bar{l}_{j-1}}\sum\limits_{\forall \bar{y}_{j-1} } E(Y_{j}|\bar{H}_{j-1} , \eta ) \prod\limits_{k=1}^{j-1} E(Y_k| \bar{H}_{k-1}, \eta ) P(L_k=l_k | \bar{H}_{k-1} ) f(\eta _i) \partial \eta _i . \end{aligned}$$

The G-computation is a generalization of covariate standardization in the longitudinal setting with time-dependent exposure to express the marginal causal effect when the identifiability assumptions are satisfied [26]. The G-computation defines the population-average (i.e. marginal) causal contrast with respect to treatment and visit conditioned on baseline covariates and random effect. We used the G-computation to estimate the marginal quantity of interest (i.e. treatment effect) in marginal structural model.

### Data generation

**Fig. 1:**
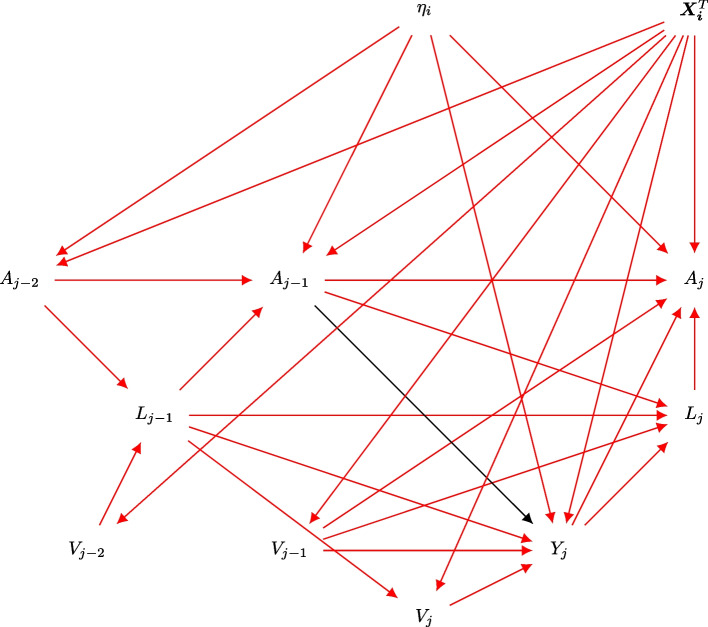
Directed cyclic graph in the presence latent subject-specific confounder with treatment-confounder and visit-confounder feedback (arrows are not drawn for vertices in time-interval j-1 and j-2)

In the conditional data generating mechanism, the most recent values for visit $V_{j-1}$, confounder $L_{j-1}$ and past treatment $A_{j-1}$ are assumed to influence the longitudinal outcome $Y_{j}$. The inter-dependencies in the collection of longitudinal information (with respect to discrete time-intervals) give rise to treatment-confounder feedback and visit-confounder feedback [27, 28]. The treatment-confounder feedback (i.e. $\cdot \cdot \cdot \rightarrow A_{j-1} \rightarrow L_j \rightarrow A_j \rightarrow \cdot \cdot \cdot$) and visit-confounder feedback (i.e. $\cdot \cdot \cdot \rightarrow V_{j-1} \rightarrow L_j \rightarrow V_{j+1} \rightarrow \cdot \cdot \cdot$) are defined over discrete time-intervals (as shown in Fig. 1).

The baseline covariates $\varvec{X}_i^T$ are assumed to be measured as

$$\begin{aligned} \varvec{X}_i^T = (X_{i1}, X_{i2}, X_{i3}) = \left( exp\left( \frac{U_{i1}}{2}\right) , \frac{U_{i1}}{1+exp(U_{i2})}, \frac{U_{i1} U_{i3}}{25} \right) \end{aligned}$$

where $\varvec{U_i^T}= (U_{i1}, U_{i2}, U_{i3})$ is a vector of unobserved covariates independently sampled from multivariate standard normal distribution. The outcome $Y_{ij}$ is generated as

$$\begin{aligned} \mathbb {E}(Y_{ij}| {A}_{j-1},V_{j-1}, L_{j-1}, \varvec{X}_i^T, \eta _i)= & {} \theta _0 + \theta _1 A_{j-1} + \theta _2 V_{j-1} + \theta _3 L_{j-1} \\{} & {} +\theta _4 X_{1} + \theta _5 X_{2} + \theta _6 X_{3} + \theta _{7} \eta _i. \end{aligned}$$

The data generating mechanism for time-varying covariates $L_{ij}$, irregular visits $V_{ij}$ and treatment $A_{ij}$ are specified as

$$\begin{aligned} P(L_{ij}| V_{j-1}, Y_{j}, L_{j-1}, A_{j-1}) = expit&(\mu _0 + \mu _1 V_{j-1} + \mu _2 Y_{j} + \mu _3 L_{j-1} + \mu _4 A_{j-1} ) \\ P(V_{ij}| L_{j-1}, \varvec{X}_i^T )=expit&(\omega _0 + \omega _1 L_{j-1} +\omega _2 X_1 +\omega _3 X_{2} + \omega _4 X_{3}) \\ P(A_{ij}|V_{j-1}, Y_{j}, L_{j}, A_{j-1},\varvec{X}_i^T, \eta _i ) = expit&(\alpha _0 +\alpha _1 V_{j-1} + \alpha _2 Y_{j} + \alpha _3 L_{j} + \alpha _4 A_{j-1} \\&+ \alpha _5 X_1 + \alpha _6 X_2 + \alpha _7 X_3 + \alpha _8 \eta _i) \\ \end{aligned}$$

where $V_{j}=L_{j}=A_{j}=Y_{j}=0$ when $j<0$ and $expit(\cdot )=\frac{exp(\cdot )}{1+exp(\cdot )}$. Bernoulli distribution is used to generate the time-varying binary factors with respect to the conditional probabilities for $V_{ij}$, $L_{ij}$ and $A_{ij}$ while the outcome is assumed to be distributed using normal distribution ($Y_i \sim N(\Theta ,\sigma ^2_Y)$). The probabilities of the binary factors $(L_{ij}, A_{ij}, V_{ij})$ are described using inverse-logit link function where positive values indicate increased probability of binary factor. The latent factor $\eta _i \sim N(0,1)$ is time-invariant for each individual and acts as a common cause for time-varying treatment $A_{ij}$ and outcome $Y_{ij}$.

In the absence of visit (i.e. $V_{ij}=0$), we assume the continuation of previous treatment $A_{ij}$ and previous covariate $L_{ij}$, and a change in treatment and covariate is only observed in the presence of visit. Moreover, we specify the outcome $Y_{ij}$ to be missing in the absence of visit. Using the conditional data generating mechanism, the joint distribution is expressed using the causal Markov factorization as

$$\begin{aligned} f(\bar{V}_{ij},\bar{Y}_{ij},\bar{L}_{ij}, \bar{A}_{ij},\eta _{ij})=\prod\limits_{i=1}^n \prod\limits_{j=1}^{m_i}{} & {} P(V_{ij}| L_{j-1}, \varvec{X}_i^T ) f(Y_{ij}| {A}_{j-1},V_{j-1}, L_{j-1}, \varvec{X}_i^T, \eta _i)^{{1}(V_{ij}=1)} \\ {}{} & {} P(L_{ij}| V_{j-1}, Y_{j}, L_{j-1}, A_{j-1}) \\ {}{} & {} P(A_{ij}|V_{j-1}, Y_{j}, L_{j}, A_{j-1},\varvec{X}_i^T, \eta _i ) \\ {}{} & {} P(X_{i1}) P(X_{i2}) P(X_{i3}) f(\eta _{ij}). \end{aligned}$$

The data generating mechanism in the presence of visit and unmeasured (latent) factor $\eta _i$ is described using a directed acyclic graphs in Fig. 1 where red edges denote associations, and black edges denote causal relationship between treatment and outcome.

### Estimation procedures

The g-computation formula (in the absence and presence of unmeasured confounder) is used to obtain the true values of marginal treatment effects (see Appendix Section). Since the time-varying treatment $A_j$ and visit $V_j$ are statistically endogenous in observational studies, the stabilized inverse probability treatment weights (sIPTW), stabilized inverse probability visit weights (sIPVW) and joint stabilized inverse probability weights (sIPTW $\times$ sIPVW) with calibration restrictions (described in Section 2.4) are used to estimate the causal effect. The correct specification of the treatment model, visit model and marginal outcome model (with the exclusion of time-dependent confounders) are used in relation to the data generating mechanism. Four simulation scenarios are considered in the simulation study:

- **Scenario 1:** absence of irregular visits (i.e. $\bar{V}_j=\mathbb {\bar{1}}$) and absence of unmeasured confounder $\eta _i$. The estimation of marginal treatment effect is performed using sIPTW and calibrated IPTW with the constraint for [a] orthogonality between treatment residual $(A_{ij} - \hat{e}_{ij}^A)$ and time-dependent covariates $L_{ij}$, [b] average treatment weights equal to one at each time interval.
- **Scenario 2:** absence of irregular visits and presence of unmeasured confounder $\eta _i$. The estimation of marginal treatment effect is performed using sIPTW and calibrated IPTW estimator with the constraint for [a], [b] and [c] covariate balance with respect to time-invariant latent confounder restriction.
- **Scenario 3:** presence of irregular visits and absence of unmeasured confounder $\eta _i$. The estimation of marginal treatment effect is performed using sIPTW, calibrated IPTW, sIPVW and calibrated IPVW estimators with the constraint for [a] orthogonality between treatment residual $(A_{ij} - \hat{e}_{ij}^A)$ (or visit residual $V_{ij}-\hat{e}_{ij}^V$) and time-dependent covariates $L_{ij}$, [b] average treatment weights equal to one at each time interval.
- **Scenario 4:** presence of irregular visits and presence of unmeasured confounder $\eta _i$. The estimation of marginal treatment effect is performed using sIPTW, calibrated IPTW, sIPVW and calibrated IPVW estimators with the constraint for [a], [b], [c] covariate balance with respect to time-invariant latent confounder restriction.

We implemented the constrained optimization of the cumulative-time product weights using the Barzilai-Borwein (BB) method [29]. After the estimation of stabilized and calibrated weights, the marginal causal effect of treatment is estimated using the pseudo-likelihood function of generalized estimating equations with AR(1) working correlation structure (geeglm function in R) with stabilized weights and calibrated weights. Based on the data generating mechanism, the correct specification of the marginal structural models for four scenarios were fitted as

$$\begin{aligned} E(Y_j^{\bar{a},\bar{v}}|X)= \left\{ \begin{array}{ll} \psi _0 + \psi _1 a_{j-1} + \psi _2 X_1 + \psi _3 X_2 + \psi _4 X_3 &{} \text {for scenario 1 and 2}\\ \psi _0 + \psi _1 a_{j-1} + \psi _2 v_{j-1} + \psi _3 X_1 + \psi _4 X_2 + \psi _5 X_3 &{} \text {for scenario 3 and 4.}\\ \end{array} \right. \end{aligned}$$

The absence of irregular visits in scenario 1 and 2 led to the exclusion of $V_{j-1}$ in the equation above. We also applied a naïve estimator by fitting unweighted generalized estimating equations with AR(1) working correlation structure. The naïve estimator was used as a reference to determine the extent of bias introduced by measured confounding, selecting bias (due to irregular visit) and unmeasured subject-specific confounding within simulated scenarios.

### Simulation parameters

Table 1 describes the simulation parameters for the data generating mechanism described in Fig. 1. The performance of inverse probability weight-based estimators is assessed using relative bias, Monte Carlo error (MCE), root mean square error (rMSE) and coverage probability. Relative bias is estimated as the average difference between an estimator and the true parameter value. MCE is defined as the standard deviation of an estimator while the rMSE is the square root sum of bias squared and MCE squared. The coverage probability was estimated as the proportion of times when the 95% confidence interval contained the true parameter value. The simulation study is repeated for 1,000 replications using a sample size $N=100$ with ten discrete time intervals.

**Table 1:** Simulation parameters for irregular visits, confounder, treatment and longitudinal outcome

Description	Simulation parameters
$Y_{ij}:$ Longitudinal outcome^a^	$\theta _0=9;\theta _1=-0.3;\theta _2=-0.1$; $\theta _3=0.5$;
	$\theta _4=\theta _5=\theta _6=0.5$; ($\theta _{7}=0.1$); $\sigma ^2_y=3$
$L_{ij}:$ Time-dependent confounder	$\mu _0=0;\mu _1=0.1; \mu _2=0$; $\mu _3=0$; $\mu _4=0.1$
$V_{ij}:$ Irregular visits^b^	$\omega _0=2 [16];\omega _1=0.1;\omega _2=0.1; \omega _3=0.1$; $\omega _4=0.1$
$A_{ij}:$ Time-dependent treatment^a^	$\alpha _0=0;\alpha _1=0.1$; $\alpha _2=0.1$ ; $\alpha _3=0.1$;
	$\alpha _4=0$; $\alpha _5=0.3$; $\alpha _6=0.3$; $\alpha _7=0.6$; ($\alpha _8=0.1$)
*N* : number of subjects	100
*t* : number of discrete time-points	10
*r* : number of replicates	1000

^a^(.) denotes the coefficient for unmeasured confounder

^b^[.] denotes the coefficient for absence of irregular visits

### Results

The results of the simulation study are presented for the four scenarios in which the presence or absence of irregular visits and time-invariant latent confounder is considered. The results are summarized using the performance metrics of relative bias, MCE, rMSE, coverage probabilities and successful convergence of calibrated weights, as shown in Table 2.

#### Scenario 1: regular visits, no unmeasured confounder

We assessed the performance of sIPTW and calibrated IPTW in the absence of irregular visits and unmeasured confounder. An improvement in bias was observed for calibrated IPTW in relation to sIPTW. The MCE and rMSE for calibrated weights was smaller than the stabilized weights, and the coverage probabilities were close to 95% nominal rate. The correlation between stabilized weights and calibrated weights ranged from 0.986 to 0.999. Successful convergence of calibrated weights was observed for all 1,000 replicates.

#### Scenario 2: regular visits, unmeasured confounder

In this scenario, we assessed the performance of sIPTW and calibrated IPTW in the absence of irregular visits, and in the presence of unmeasured confounder. Nominal coverage rates close to 95% were observed for marginal structural models with sIPTW and calibrated IPTW. The calibrated weights had smaller mean bias than stabilized weights. The calibrated weights also had smaller MCE and rMSE than stabilized weights. The correlation between stabilized weights and calibrated weights ranged from 0.929 to 0.999. Successful convergence was observed for 959 out of 1,000 replicates.

**Table 2:** Finite sample properties using Monte Carlo simulation study

Estimator	Effect	$\psi$	Estimate	Bias	R. Bias(%)	MCE^a^	rMSE^b^	$\alpha$-level
**Scenario 1: No irregular visits, no unmeasured confounder**								
Naïve	$A_{j-1}$	-0.3	-0.274	0.0260	8.67%	0.2966	0.2978	0.939
sIPTW	$A_{j-1}$	-0.3	-0.2796	0.0204	6.8%	0.3183	0.3189	0.944
cIPTW	$A_{j-1}$	-0.3	-0.2842	0.0158	5.27%	0.3071	0.3075	0.943
**Scenario 2: No irregular visits, unmeasured confounder**								
Naïve	$A_{j-1}$	-0.3	-0.2642	0.0358	11.93%	0.3484	0.3502	0.936
sIPTW	$A_{j-1}$	-0.3	-0.2839	0.0161	5.37%	0.3181	0.3185	0.928
cIPTW	$A_{j-1}$	-0.3	-0.2909	0.0091	3.03%	0.3138	0.3139	0.940
**Scenario 3: Irregular visits, no unmeasured confounder**								
Naïve	$A_{j-1}$	-0.3	-0.2678	0.0322	10.73%	0.3421	0.3436	0.927
sIPTW	$A_{j-1}$	-0.3	-0.2719	0.0281	9.37%	0.3332	0.3343	0.934
sIPVW	$A_{j-1}$	-0.3	-0.2776	0.0224	7.47%	0.3142	0.3150	0.940
sIPTW$\times$sIPVW	$A_{j-1}$	-0.3	-0.2859	0.0141	4.7%	0.3292	0.3295	0.935
cIPTW$\times$cIPVW	$A_{j-1}$	-0.3	-0.3029	-0.0029	-0.97%	0.3341	0.3341	0.944
**Scenario 4: Irregular visits, unmeasured confounder**								
Naïve	$A_{j-1}$	-0.3	-0.2612	0.0388	12.93%	0.3397	0.3419	0.921
sIPTW	$A_{j-1}$	-0.3	-0.2672	0.0328	10.93%	0.3317	0.3333	0.938
sIPVW	$A_{j-1}$	-0.3	-0.2692	0.0308	10.27%	0.3162	0.3177	0.932
sIPTW$\times$sIPVW	$A_{j-1}$	-0.3	-0.2777	0.0223	7.43%	0.3294	0.3301	0.940
cIPTW$\times$cIPVW	$A_{j-1}$	-0.3	-0.2932	0.0068	2.27%	0.2934	0.2934	0.947

$\psi =$ marginal causal effect

sIPT(V)W= stabilized inverse probability treatment (visit) weights

cIPT(V)W= calibrated inverse probability treatment (visit) weights

^a^MCE= Monte Carlo Error (standard deviation of Monte Carlo estimator)

^b^rMSE = root Mean Square Error= $\sqrt{Bias^2+Var}$

#### Scenario 3: irregular visits, no unmeasured confounder

In this scenario, we assessed the performance of stabilized and calibrated variants of treatment and visit weights in the presence of irregular visits and absence of unmeasured confounder. The coverage probabilities were close to the expected 95% nominal rate for the four estimators: (i) sIPTW, (ii) sIPVW, (iii) joint sIPTW and sIPVW, (iv) and calibrated IPTW and IPVW. Smaller bias was observed for calibrated weights when compared to the stabilized weights. The correlation between stabilized weights and calibrated weights ranged from 0.25 to 0.99, and successful convergence was observed for all 1,000 replicates.

#### Scenario 4: irregular visits, unmeasured confounder

In this scenario, we assessed the performance of stabilized and calibrated variants of treatment and visit weights in the presence of irregular visits and unmeasured confounder. The coverage probabilities were close to the expected 95% nominal rate for all estimators. A reduction in bias was observed for calibrated IPTW and IPVW estimators when compared with sIPTW and sIPVW estimators. The correlation between stabilized weights and calibrated weights ranged from 0.24 to 0.99, and successful convergence was recorded for 966 out of 1,000 replicates.

## Application: emulating randomized experiment using EHRs

Electronic health records (EHRs) can serve as a complete lifetime record of a patient’s longitudinal health trajectory, and they can be collected from different sources including hospitals, specialist clinics, primary care providers, pharmacies, and laboratories. University of Toronto practice based research network (UTOPIAN) collects de-identified patient-level medical information from EHRs of primary care practices across the Greater Toronto region [30, 31]. The EHRs in UTOPIAN database contains patient-level demographics, medical diagnosis, procedures, medications, immunization, laboratory test results, vital signs and risk factors. Even though the UTOPIAN repository is a rich source of de-identified patient-level medical information, it is prone to many sources of bias including informed presence of patients due to acute onset of new medical ailment or the management of pre-existing chronic condition. In our context, we used the irregular visits in longitudinal follow-up to describe the informed presence of diabetes patients in primary care.

### Glucose lowering medications

Diabetes is one of the most common chronic conditions in which the body cannot properly use insulin produced by pancreas (Type II). Hemogloblin A1c (HbA1c) is an important glycemic marker to reduce the incidence of diabetes-related complications and mortality [32]. More than 90% of type II diabetes patients eventually require more than metformin monotherapy to achieve their target for optimal glucose control. Dual or combination therapy may become necessary when metformin is insufficient. The second-line options for glucose management include sodium-glucose co-transporter 2 inhibitors (SGLT-2i) and dipeptidyl peptidase-4 inhibitors (DPP-4i) medications. SGLT-2i medications are associated with reduction in cardiovascular outcomes and decreased progression of renal disease [6], while DPP-4i medications are known to have highly favourable tolerability among elderly patients [33]. We assess the effectiveness of SGLT-2i and DPP-4i drugs using HbA1c as the glycemic marker among patients with type II diabetes. We assume intention-to-treat design for time-varying treatment assignment where the analysis are based on the treatment assignment (i.e. drug prescription) rather than the treatment eventually received (i.e. drug dispensation).

**Table 3:** A summary of target trial to estimate the change in HbA1c^a^ among type II diabetes patients

Protocol component	Description
Follow-up period	Study follow-up starts on January 01, 2018 and terminated on September 30, 2021. Patient follow-up defined with eligibility and censoring criteria.
Exclusion criteria	Exclude patients with three year look-back window for SGLT-2i^b^ and DPP-4i^c^ prescriptions with respect to the start of the study period (January 01 2018).
Eligibility criteria	At least 18 years old patients with diabetes and elevated HbA1c $(\ge 8.5 \%)$.
Censoring criteria	Administratively censored on September 30, 2021 or mid-calendar year (June 30) when deceased year is recorded.
Treatment strategy	SGLT-2i medication v.s. standard care (i.e. without SGLT-2i prescriptions), and DPP-4i medication v.s. standard care (i.e. without DPP-4i prescriptions).
Assignment procedures	Participants randomly assigned to either treatment strategy.
Outcome	Repeat-measures HbA1c (in %).
Causal contrast of interest	Cumulative SGLT-2i prescriptions v.s. standard care.
	Cumulative DPP-4i prescriptions v.s. standard care.
Analysis plan	Intention-to-treat analysis.

^a^HbA1c= Hemoglobin A1c;

^b^SGLT-2i= Sodium-Glucose co-Transporters 2 Inhibitor;

^c^DPP-4i = Dipeptidyl Peptidase-4 Inhibitor

### Cohort generation

We emulate a randomized experiment using an observational setting, and describe several elements of emulating the target trial in Table 3. Patients are enrolled in the longitudinal cohort from January 01 2018 when the following conditions are satisfied: (i) patient is at least 18 years of age; (ii) patient has a clinical indication for diabetes [34]; (iii) HbA1c $\ge 8.5 \%$ is recorded within the study period. Patient follow-up starts when these eligibility criteria (i)-(iii) are met. Patients are administratively censored on September 30 2021 or censored at mid-calendar (June 30) when deceased year is recorded. The enrollment period is terminated on January 01 2020 while the study follow-up is terminated on September 30 2021. Once the patient is enrolled in the cohort, three month time-intervals are generated under the assumption that the patient regularly visits the clinic on quarterly basis (i.e. every 3 months) as recommended by Diabetes Canada guidelines [6]. The presence of billing record for any primary care services within a quarter for a given patient indicate $V_{ij}=1$. As an example, $V_{ij}=0$ if no billing record is available within an index quarter and $V_{ij}=1$ if patient has one or more billing records within an index quarter. The time-dependent exposures $A_{ij}^\prime$ and $A_{ij}^{\prime \prime }$ (prescription for SGLT-2i and DPP-4i medications), time-dependent confounders $L_{ij}$ (co-morbidities and glucose lowering medications, respectively), and continuous outcome $Y_{ij}$ (Hemoglobin A1c) are assumed to be constant within each index quarter. In the case of multiple visits within each quarter, positive values of $A_{ij}^\prime$, $A_{ij}^{\prime \prime }$ and $L_{ij}$ take precedence while an average value of $Y_{ij}$ is computed for each patient within each quarter. Once the positive indication for a co-morbidity is recorded, the patient is assumed to have the co-morbidity for the remainder of the study period. Patients who had an earlier prescription for SGLT-2i or DPP-4i medications three years prior to January 01 2018 were excluded. The three-year look back window reduced the possibility of selection bias by left truncating those individuals who initiated the secondary treatment (using SGLT-2i or DPP-4i) prior to meeting the eligibility criteria.

We assume the time-varying patient characteristics (i.e. co-morbidities, other glucose-lowering medications) as time-dependent confounders. Other glucose lowering medications $(X_{ij})$ include the use of monotherapy and combination therapy using several drug classes: (i) metformin, (ii) GLP-1, (iii) sulfonylurea, (iv) insulin, as detailed elsewhere [35]. We included several co-morbidities with disease onset date as time-dependent covariates: (i) chronic obstructive pulmonary disease (COPD), (ii) depression, (iii) dyslipidemia, (iv) hypertension, (v) osteoarthritis, (vi) chronic kidney disease, as detailed elsewhere [34].

### Marginal structural model

The effectiveness of glucose lowering medications was assessed among diabetes patients who were prescribed SGLT-2i and/or DPP-4i medications during the study period. Longitudinal marginal structural models using generalized estimating equations (AR-1 correlation) with stabilized and calibrated weights were used to generate covariate balance with respect to treatment assignment (i.e. SGLT-2i and DPP-4i medications) and irregular visits. We fitted the following marginal structural model:

7 $$\begin{aligned} \nonumber E(Y_{ij}^{\bar{a},\bar{v}}|H_{j-1}^*) = \theta _0{} & {} + \theta _1 \times \text {age group}_{ij} \\ \nonumber{} & {} + \theta _{2} \times \text {sex}_{i} \\{} & {} + \theta _{3} \times \text {income quintiles}_{i} \\ \nonumber{} & {} + \theta _{4} \times \text {rurality}_{i} \\ \nonumber{} & {} + \theta _5 \times \sum\limits_{j} (\text {SGLT-2i prescription})_{ij} \\ \nonumber{} & {} + \theta _6 \times \sum\limits_{j} (\text {DPP-4i prescription})_{ij} \\ \nonumber{} & {} + \theta _7 \times \text {visit}_{ij-1} \\ \nonumber{} & {} + \theta _8 \times \text {baseline HbA1c}_{i} \end{aligned}$$

where $\sum _{j}$ denotes the cumulative number of prescriptions for glucose lowering medications, truncated at two prescriptions. The marginal structural model did not include the time-dependent covariates $L_{ij}$ as they were accounted for using the longitudinal weights. The pseudo-populations were defined using the stabilized weights with their calibrated counterparts defined as

$$\begin{aligned} SW^{A^{\prime }}_t=\prod\limits_{j=1}^{t} \frac{ P(A^{\prime }_{j-1} |\bar{H}_{j-1}^* )}{ Pr(A^{\prime }_{j-1} |\bar{H}_{j-1} )}; \quad \quad SW^{A^{\prime }}_t(\lambda _1) = SW^{A^{\prime }}_t \times exp(K\lambda _1) \end{aligned}$$

$$\begin{aligned} SW^{A^{\prime \prime }}_t=\prod\limits_{j=1}^{t} \frac{ P(A^{\prime \prime }_{j-1} |\bar{H}_{j-1}^*)}{ Pr(A^{\prime \prime }_{j-1} |\bar{H}_{j-1} )}; \quad \quad SW^{A^{\prime \prime }}_t(\lambda _2) = SW^{A^{\prime \prime }}_t \times exp(K\lambda _2) \end{aligned}$$

$$\begin{aligned} SW^{V}_t=\prod\limits_{j=1}^{t} \frac{ P(V_{j-1} |\bar{H}_{j-1}^* )}{ Pr(V_{j-1} |\bar{H}_{j-1} )}; \quad \quad SW^{V}_t(\lambda _3) = SW^{V}_t \times exp(K\lambda _3) \end{aligned}$$

$$\begin{aligned} SW^{A^{\prime },A^{\prime \prime }, V}_t= & {} SW^{A^{\prime }}_t \times SW^{A^{\prime \prime }}_t \times SW^{V}_t \\ SW^{A^{\prime },A^{\prime \prime }, V}_t (\tilde{\lambda })= & {} SW^{A^{\prime }}_t(\lambda _1) \times SW^{A^{\prime \prime }}_t(\lambda _2) \times SW^{V}_t(\lambda _3) \end{aligned}$$

where $\bar{A}^{\prime }$ denotes treatment for SGLT-2i medications and $\bar{A}^{\prime \prime }$ denotes treatment for DPP-4i medications, and $\tilde{\lambda }=\{\lambda _1 , \lambda _2 , \lambda _3 \}$. The stabilized weights with respect to the SGLT-2i medications, DPP-4i medications, and irregular visits were used to construct the pseudo-populations. At last, the product weights of SGLT-2i medications, DPP-4i medication and irregular visits were calibrated to balance the time-dependent covariate distributions. The calibration restrictions included the balancing conditions for the orthogonality constraint (i.e. $(A_{ik} - \hat{e}_{ik}^A) {\perp \! \! \! \perp } \tilde{L}_{ik-1}$ and $(V_{ik} - \hat{e}_{ik}^A) {\perp \! \! \! \perp } \tilde{L}_{ik-1}$), and unity constraints (i.e. $E(SW_{j})=1 \ \forall j$). We truncated the stabilizing and calibrated weight functions at 1% and 99% quantiles to improve the estimation of the marginal effects [36].

### Effectiveness of glucose lowering medications

**Fig. 2:**
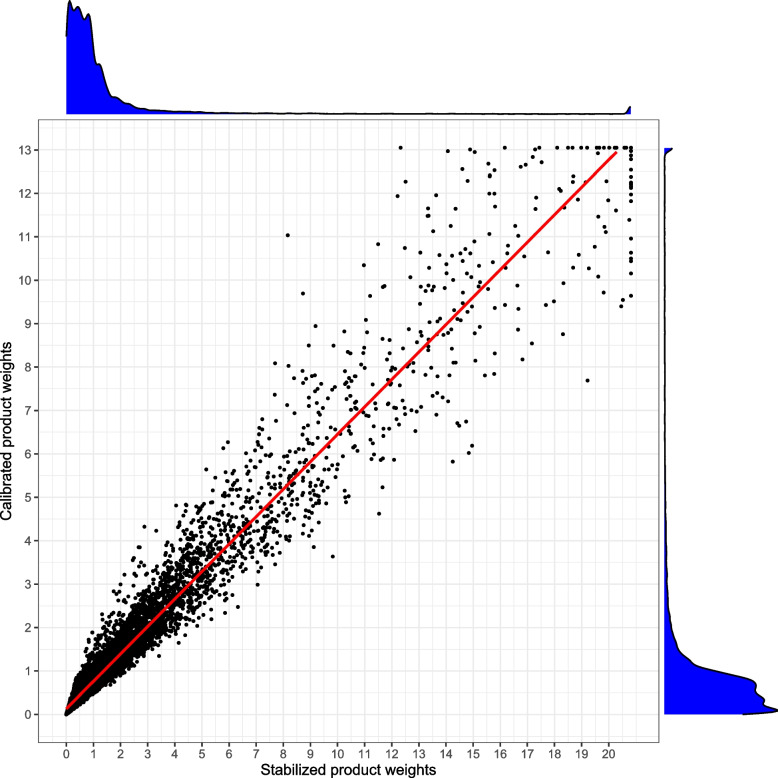
Scatter plot of stabilized and calibrated product weights

**Fig. 3:**
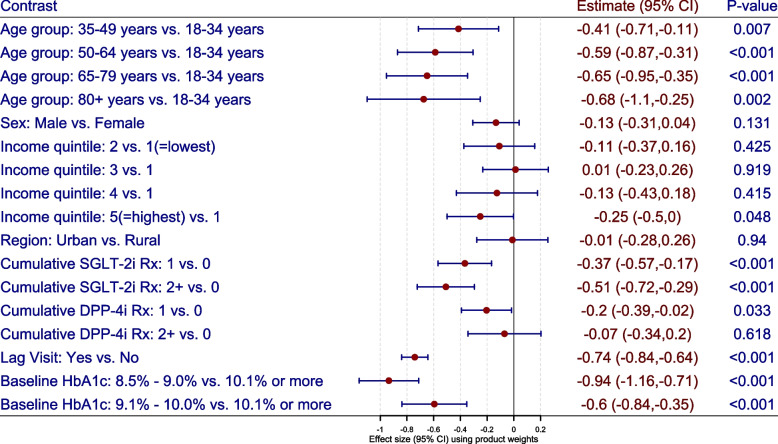
Marginal structural model using the stabilized product weights

**Fig. 4:**
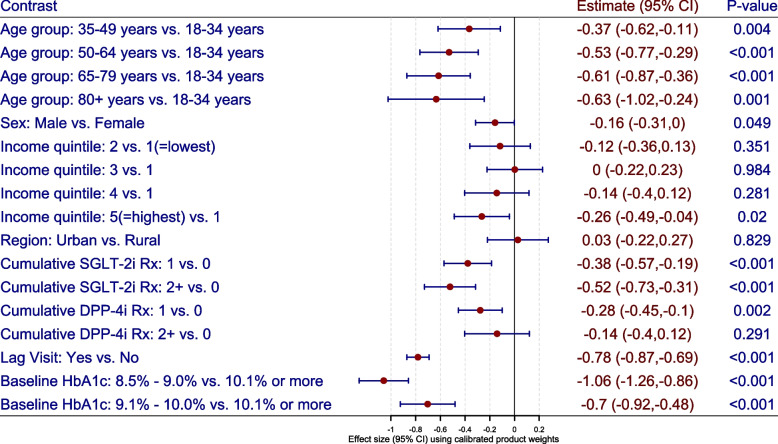
Marginal structural model using the calibrated product weights

We describe the effectiveness of glucose lowering medications among diabetes patients with elevated HbA1c (i.e. $\ge 8.5 \%$) using the stabilized (product) weights and the calibrated weights. The pseudo-population characterized using the product weights (Fig. 3) and calibrated weights (Fig. 4) were used to describe the net change in mean HbA1c with respect to the patient demographics, geographical characteristics and treatment assignment. The stabilized (product) weights ranged from 0.003 to 20.808 with mean value of 1.381 while the calibrated weights ranged from 0.003 to 13.045 with mean value of 1.002 (see Fig. 2). The correlation between stabilized and calibrated weights was 0.985.

Using the calibrated weights, we note that older patients have lower mean HbA1c than younger patients (e.g. $65-79$ years vs. $18-34$ years: $-0.63\%$ (95% CI: $-1.02\%$ to $-0.24\%$, *P*-value $< 0.001$)). The mean HbA1c is lower among male patients than female patients ($-0.16\%$ (95% CI: $-0.31\%$ to $0.00\%$, *P*-value $=0.049$)). The mean HbA1c is lower among patients residing in highest income quintiles than lowest income quintiles ($-0.26$ % (95% CI: $-0.49\%$ to $-0.04\%$, *P*-value $=$ 0.02)). We note that the mean HbA1c is reduced with glucose lowering medications (SGLT-2i and DPP-4i). For example, the mean HbA1c is reduced by $-0.38\%$ (95% CI: $-0.57\%$ to $-0.19\%$, *P*-value $<0.001$) with a single prescription of SGLT-2i medication while it is reduced by $-0.28\%$ (95% CI: $-0.45\%$ to $-0.10\%$, *P*-value$<0.001$) with a single prescription of DPP-4i medication. A reduction of $-0.78\%$ (95% CI: $-0.87\%$ to $-0.69\%$) in mean HbA1c is observed with primary care visit, indicating improved regulation of blood glucose. Similar findings were observed for stabilized weights (when compared to calibrated weights) as the inference did not change drastically (see Figs. 3 and 4).

## Discussion

The longitudinal data in EHRs feature several methodological complexities. For example, the irregular visits in EHRs are often recorded in which the longitudinal outcome is measured sporadically across time-intervals, and it may depend on patients’ observed history (i.e. missing at random) [5]. Furthermore, the presence of time-invariant latent confounders in EHRs may generate biased estimation of treatment effect [37].

In this article, we proposed calibrated weights to estimate the effect of time-dependent treatment on longitudinal outcomes with irregular visits and with unmeasured confounding. We derived the calibrated restrictions for subject-specific latent confounder which acts as a common cause for treatment and outcome. In our simulation study, the effect of time-varying treatment propagated towards the longitudinal outcome in multiple pathways: (i) direct effect of treatment on outcome, (ii) indirect effect of treatment through the time-varying covariate, (iii) indirect effect of treatment through the irregular visits. The proposed weights created a pseudo-population in which the irregular visits were exogenous (using the visit weights), and in which the treatment groups were exchangeable (using the treatment weights). Marginal structural model with inverse probability weights with respect to treatment accounted for confounding bias in treatment-confounder feedback while the inverse probability weights with respect to irregular visit accounted for selection bias in visit-confounder feedback [8]. An improvement in finite sample properties (e.g. reduced bias) was observed with the application of calibrated weights. We further employed the calibrated weights to assess the effect of cumulative prescription of glucose lowering medications (SGLT-2i and DPP-4i) on Hemoglobin A1c.

Similar to calibrated weights, Imai and Ratkovic [24] proposed a methodology for covariate balancing weights which improved performance by reducing the mean square error under correct and incorrect model misspecification. Covariate balancing weights are computationally intensive and may only accommodate small number of follow-up visits and covariates in longitudinal settings due to increase in the total number of moment conditions for weight estimation [13]. Even when the causal identifiability assumptions hold, biased estimation of average treatment effect is possible for longitudinal marginal structural models or g-computation when the parametric models are misspecified. In the earlier literature [20] and in this article, the calibrated restrictions on inverse probability weights improved the estimation error under finite sample when compared to maximum likelihood estimation.

Young et al. [38] discretized continuous-time and used pooled logistic regression with short intervals to estimate the causal effect of treatment on longitudinal outcome with rare occurrence. If we consider binary repeated-measures outcome (e.g. elevated Hemoglobin A1c) then we may specify short discrete time intervals with negligible event rate where the time-dependent treatment is influenced with respect to the observed history. Varying the length of time-intervals may also describe the extent of irregularity in follow-up visits [7]. Furthermore, in this article, attention was limited to irregular visits where the entire study population was assumed to follow a single type of homogeneous process. As alluded by Neuhaus et al. [39], a more realistic approach could be to consider a combination of regular and irregular visits in the longitudinal cohort using the outcome-visit dependency. One approach to incorporate this could be to cluster the patient profiles (with respect to irregularities in visits) using latent class analysis prior to assessing the effectiveness of glucose lowering medications [32].

In this article, we made the assumption of sequential positivity with respect to the treatment and irregular visits. Sequential positivity assumption may become implausible in some situations when the clinical profile of a patient precludes them from receiving SGLT-2i or DPP-4i medications, or precludes them from visiting the primary care clinic for some time-intervals. As an extension when the sequential postivity assumption is unrealistic, we may employ doubly robust estimators to reduce bias and imprecision due to large weights. Doubly robust estimators may improve the robustness against model misspecification when either the treatment model or the counterfactual outcome model is misspecified [40]. For example, Pullenayegum and Feldman [41] described the doubly robust estimator which is robust to the misspecification of the visit model (using the inverse intensity weights) and imputation model (using increment estimator). Future research may focus on the extension of calibrated estimators to accommodate for the misspecification of treatment or outcome models using doubly robust estimation [20]. The proposed estimators with calibrated restrictions also relied on the assumption of sequential exchangeability where all potential confounders are measured at each time-interval (i.e. no unmeasured time-variant confounders). In a situation where the sequential exchangeability assumption is violated, Coulombe et al. [42] and Streeter et al. [43] describe sensitivity analysis to assess the extent to which unmeasured confounding can affect the marginal effect of the treatment. As an extension to this article, sensitivity analysis may become necessary if we consider unmeasured time-dependent covariates in longitudinal settings.

### Conclusion

We demonstrated the application of calibrated weights to assess the effect of irregular visits and to assess the efficacy of glucose lowering medications (SGLT-2i and DPP-4i) on the longitudinal trajectory of Hemoglobin A1c. The empirical results showed a reduction in mean Hemoglobin A1c with cumulative prescriptions of SGLT-2i and DPP-4i medications using primary care EHRs. These findings support the earlier results from a meta-analysis in which SGLT-2i medications had stronger efficacy than DPP-4i medications [44]. In general, stronger efficacy of one treatment (e.g. SGLT-2i medications) in reducing Hemoglobin A1c is not a clinical rationale for selecting a particular treatment (e.g. DPP-4i medications). Instead, the treatment for glycemic control in type II diabetes patients is often intertwined with several clinical characteristics including weight change, blood pressure, cardiovascular profile, renal profile, and other safety profiles [45].

## Supplementary Information

**Additional file 1.** Appendix.

## Data Availability

The research ethics approval for the use of Electronic health records (EHRs) data does not permit making the data publicly available. Researchers interested in accessing EHR data from the Data Safe Haven for research can apply to do so at: https://www.dfcm.utoronto.ca/getting-utopian-support.

## References

[1] Hernán MA, Robins JM (2016). Using big data to emulate a target trial when a randomized trial is not available. Am J Epidemiol..

[2] Goldstein BA, Phelan M, Pagidipati NJ, Peskoe SB (2019). How and when informative visit processes can bias inference when using electronic health records data for clinical research. J Am Med Inform Assoc..

[3] Lin H, Scharfstein DO, Rosenheck RA (2004). Analysis of longitudinal data with irregular, outcome-dependent follow-up. J R Stat Soc Ser B Stat Methodol..

[4] McCulloch CE, Neuhaus JM, Olin RL (2016). Biased and unbiased estimation in longitudinal studies with informative visit processes. Biometrics..

[5] Pullenayegum EM, Lim LS (2016). Longitudinal data subject to irregular observation: A review of methods with a focus on visit processes, assumptions, and study design. Stat Methods Med Res..

[6] Lipscombe L, Booth G, Butalia S, Dasgupta K, Eurich DT, Goldenberg R (2018). Pharmacologic glycemic management of type 2 diabetes in adults. Can J Diabetes..

[7] Lokku A, Lim LS, Birken CS, Pullenayegum EM (2020). Summarizing the extent of visit irregularity in longitudinal data. BMC Med Res Methodol..

[8] Hernán MA, McAdams M, McGrath N, Lanoy E, Costagliola D (2009). Observation plans in longitudinal studies with time-varying treatments. Stat Methods Med Res..

[9] Tan KS, French B, Troxel AB (2014). Regression modeling of longitudinal data with outcome-dependent observation times: extensions and comparative evaluation. Stat Med..

[10] Gasparini A, Abrams KR, Barrett JK, Major RW, Sweeting MJ, Brunskill NJ (2020). Mixed-effects models for health care longitudinal data with an informative visiting process: A Monte Carlo simulation study. Statistica Neerlandica..

[11] Hernán MA, Brumback B, Robins JM (2001). Marginal structural models to estimate the joint causal effect of nonrandomized treatments. J Am Stat Assoc..

[12] Hernán MA, Robins JM. Causal inference. Boca Raton: Chapman & Hall/CRC, forthcoming; 2022.

[13] Yiu S, Su L. Joint calibrated estimation of inverse probability of treatment and censoring weights for marginal structural models. Biometrics. 2022;78(1):115–27. Wiley Online Library. 10.1111/biom.13411 PMC7612568 33247594

[14] Robins JM, Tsiatis AA (1991). Correcting for non-compliance in randomized trials using rank preserving structural failure time models. Commun Stat Theory Methods..

[15] Tan Z (2020). Regularized calibrated estimation of propensity scores with model misspecification and high-dimensional data. Biometrika..

[16] Zubizarreta JR (2015). Stable weights that balance covariates for estimation with incomplete outcome data. J Am Stat Assoc..

[17] Yiu S, Su L. Joint calibrated estimation of inverse probability of treatment and censoring weights for marginal structural models. arXiv preprint arXiv:1806.05144. 2018. 10.1111/biom.13411 PMC7612568 33247594

[18] Propensity score weighting for causal inference with clustered data. J Causal Inference. 2018;6(2). De Gruyter.

[19] Imai K, King G, Stuart EA (2008). Misunderstandings between experimentalists and observationalists about causal inference. J R Stat Soc A Stat Soc..

[20] Yiu S, Su L (2018). Covariate association eliminating weights: a unified weighting framework for causal effect estimation. Biometrika..

[21] Hernán MA, Robins JM (2006). Estimating causal effects from epidemiological data. J Epidemiol Community Health..

[22] Kang JD, Schafer JL (2007). Demystifying double robustness: A comparison of alternative strategies for estimating a population mean from incomplete data. Stat Sci..

[23] Tan Z (2010). Bounded, efficient and doubly robust estimation with inverse weighting. Biometrika..

[24] Imai K, Ratkovic M. Covariate balancing propensity score. J R Stat Soc Ser B (Statistical Methodology). 2014;76(1):243–63. Wiley Online Library.

[25] Robins JM, Greenland S, Hu FC (1999). Estimation of the causal effect of a time-varying exposure on the marginal mean of a repeated binary outcome. J Am Stat Assoc..

[26] Daniel RM, Cousens S, De Stavola B, Kenward MG, Sterne J (2013). Methods for dealing with time-dependent confounding. Stat Med..

[27] Zeger SL, Liang KY. Feedback models for discrete and continuous time series. Stat Sin. 1991:51–64.

[28] Robins J (1989). The control of confounding by intermediate variables. Stat Med..

[29] Varadhan R, Gilbert P (2009). BB: An R package for solving a large system of nonlinear equations and for optimizing a high-dimensional nonlinear objective function. J Stat Softw..

[30] Birtwhistle R, Keshavjee K, Lambert-Lanning A, Godwin M, Greiver M, Manca D (2009). Building a pan-Canadian primary care sentinel surveillance network: initial development and moving forward. J Am Board Fam Pract..

[31] Tu K, Greiver M, Kidd MR, Upshur R, Mullin A, Medeiros H, et al. The University of Toronto Family Medicine Report. Toronto: Department of Family and Community Medicine; 2019. (ISBN: 978 1 999 0809 0 7).

[32] Luo M, Lim WY, Tan CS, Ning Y, Chia KS, van Dam RM (2017). Longitudinal trends in HbA1c and associations with comorbidity and all-cause mortality in Asian patients with type 2 diabetes: a cohort study. Diabetes Res Clin Pract..

[33] Karagiannis T, Paschos P, Paletas K, Matthews DR, Tsapas A. Dipeptidyl peptidase-4 inhibitors for treatment of type 2 diabetes mellitus in the clinical setting: systematic review and meta-analysis. BMJ. 2012;344. 10.1136/bmj.e1369 22411919

[34] CPCSSN. Case Definitions: Canadian Primary Care Sentinel Surveillance Network (CPCSSN), Version 2020-Q2. 2020. http://cpcssn.ca/wp-content/uploads/2020/10/CPCSSN-Case-Definitions-2020-Q2-1.pdf. Accessed 15 May 2020.

[35] Greiver M, Havard A, Bowles JK, Kalia S, Chen T, Aliarzadeh B (2021). Trends in diabetes medication use in Australia, Canada, England, and Scotland: a repeated cross-sectional analysis in primary care. Br J Gen Pract..

[36] Xiao Y, Moodie EE, Abrahamowicz M (2013). Comparison of approaches to weight truncation for marginal structural Cox models. Epidemiol Methods..

[37] Imai K, Kim IS (2019). When should we use unit fixed effects regression models for causal inference with longitudinal data?. Am J Polit Sci..

[38] Young JG, Hernán MA, Picciotto S, Robins JM (2010). Relation between three classes of structural models for the effect of a time-varying exposure on survival. Lifetime Data Anal..

[39] Neuhaus JM, McCulloch CE, Boylan RD (2018). Analysis of longitudinal data from outcome-dependent visit processes: Failure of proposed methods in realistic settings and potential improvements. Stat Med..

[40] Bang H, Robins JM (2005). Doubly robust estimation in missing data and causal inference models. Biometrics..

[41] Pullenayegum EM, Feldman BM (2013). Doubly robust estimation, optimally truncated inverse-intensity weighting and increment-based methods for the analysis of irregularly observed longitudinal data. Stat Med..

[42] Coulombe J, Moodie EE, Platt RW, Renoux C. Estimation of the marginal effect of antidepressants on body mass index under confounding and endogenous covariate-driven monitoring times. arXiv preprint arXiv:2106.14364. 2021.

[43] Streeter AJ, Lin NX, Crathorne L, Haasova M, Hyde C, Melzer D (2017). Adjusting for unmeasured confounding in nonrandomized longitudinal studies: a methodological review. J Clin Epidemiol..

[44] Wang Z, Sun J, Han R, Fan D, Dong X, Luan Z (2018). Efficacy and safety of sodium-glucose cotransporter-2 inhibitors versus dipeptidyl peptidase-4 inhibitors as monotherapy or add-on to metformin in patients with type 2 diabetes mellitus: A systematic review and meta-analysis. Diabetes Obes Metab..

[45] Scheen A (2020). Reduction in HbA1c with SGLT2 inhibitors vs. DPP-4 inhibitors as add-ons to metformin monotherapy according to baseline HbA1c: A systematic review of randomized controlled trials. Diabetes Metab.

