# Supplementary material for: Estimation of marginal structural models under irregular visits and unmeasured confounder: calibrated inverse probability weights
Source: BMC Med Res Methodol. 2023 Jan 7;23:4. doi: 10.1186/s12874-022-01831-2 (PMC9825036; doi:10.1186/s12874-022-01831-2)
Supplement: Supplementary file 1 — Additional file 1. Appendix. [file 12874_2022_1831_MOESM1_ESM.pdf]

1. APPENDIX

A. G-formula for longitudinal outcomes without latent confounder

The marginal expectation of longitudinal potential outcome $Y_j^{\bar{a},\bar{v}}$ at j^{th} visit can be noted as

$$\psi_j^{\bar{a},\bar{v}} = \mathbb{E}(Y_j^{\bar{a},\bar{v}}|X = x) \tag{S1}$$

$$= \sum_{l_{j-1}^{\bar{a},\bar{v}}} \mathbb{E}(Y_j^{\bar{a},\bar{v}}|l_{j-1}^{\bar{a},\bar{v}}, x)P(L_{j-1}^{\bar{a},\bar{v}} = l_{j-1}^{\bar{a},\bar{v}}|x) \tag{S2}$$

$$= \sum_{l_{j-1}^{\bar{a},\bar{v}}} \mathbb{E}(Y_j^{\bar{a},\bar{v}}|a_{j-1}, v_{j-1}, l_{j-1}^{\bar{a},\bar{v}}, x)P(L_{j-1}^{\bar{a},\bar{v}} = l_{j-1}^{\bar{a},\bar{v}}|a_{j-1}, v_{j-1}, x) \tag{S3}$$

$$= \sum_{y_{j-1}^{\bar{a},\bar{v}}} \sum_{l_{j-1}^{\bar{a},\bar{v}}} \mathbb{E}(Y_j^{\bar{a},\bar{v}}|a_{j-1}, v_{j-1}, l_{j-1}^{\bar{a},\bar{v}}, y_{j-1}^{\bar{a},\bar{v}}, x)P(L_{j-1}^{\bar{a},\bar{v}} = l_{j-1}^{\bar{a},\bar{v}}|a_{j-1}, v_{j-1}, y_{j-1}^{\bar{a},\bar{v}}, x)f(Y_{j-1}^{\bar{a},\bar{v}}|a_{j-1}, v_{j-1}, l_{j-1}^{\bar{a},\bar{v}}, x) \tag{S4}$$

It can be noted:

- Equation (S2) by marginalization with respect to $l_j^{\bar{a},\bar{v}}$.
- Equation (S3) by sequential randomization assumption: $\{Y_j^{\bar{a},\bar{v}}, L_j^{\bar{a},\bar{v}}\} \perp\!\!\!\perp \{A_j, V_j\}|\bar{A}_{j-1}, \bar{L}_j, \bar{V}_{j-1}, X$.
- Equation (S4) by marginalization with respect to $y_{j-1}^{\bar{a},\bar{v}}$.

By iterating, equation (S2)-(S4) on $(Y_{j-2}^{\bar{a},\bar{v}}, L_{j-2}^{\bar{a},\bar{v}}, A_{j-2}, V_{j-2}), (Y_{j-3}^{\bar{a},\bar{v}}, L_{j-3}^{\bar{a},\bar{v}}, A_{j-3}, V_{j-3}) \cdots (Y_1^{\bar{a},\bar{v}}, L_1^{\bar{a},\bar{v}}, A_1, V_1)$ and then applying the consistency assumption for $Y_j^{\bar{a},\bar{v}}$ and $L_j^{\bar{a},\bar{v}}$, we get

$$\psi_j^{\bar{a},\bar{v}} = \mathbb{E}(Y_j^{\bar{a},\bar{v}}|X = x) \tag{S5}$$

$$= \sum_{\forall \bar{l}_{j-1}} \sum_{\forall \bar{y}_{j-1}} \mathbb{E}(Y_j|\bar{a}_{j-1}, \bar{v}_{j-1}, \bar{l}_{j-1}, \bar{y}_{j-1}, x) \prod_{k=1}^{j-1} P(L_k = l_k|\bar{a}_{k-1}, \bar{v}_{k-1}, \bar{l}_{k-1}, \bar{y}_{k-1}, x)f(Y_k|\bar{a}_{k-1}, \bar{v}_{k-1}, \bar{l}_{k-1}, \bar{y}_{k-1}, x) \tag{S6}$$

$$= \sum_{\forall \bar{l}_{j-1}} \sum_{\forall \bar{y}_{j-1}} \mathbb{E}(Y_j|\bar{H}_{j-1}) \prod_{k=1}^{j-1} f(Y_k|\bar{H}_{k-1})P(L_k = l_k|\bar{H}_{k-1}) \tag{S7}$$

(S8)

where $\bar{H}_{k-1} = \{\bar{a}_{k-1}, \bar{v}_{k-1}, \bar{l}_{k-1}, \bar{y}_{k-1}, x\}$ denotes the history up to and including visit $k-1$.

B. G-formula for longitudinal outcomes with latent confounder

We extend the previous derivation of G-formula in the absence of unmeasured confounder to the G-formula marginalizing over the distribution of the unmeasured confounder as

$$\psi_j^{\bar{a},\bar{v}} = \int \sum_{\forall \bar{l}_{j-1}} \sum_{\forall \bar{y}_{j-1}} \mathbb{E}(Y_j|\bar{H}_{j-1}, \eta) \prod_{k=1}^{j-1} f(Y_k|\bar{H}_{k-1}, \eta)P(L_k = l_k|\bar{H}_{k-1}, \eta)f(\eta_i)\partial\eta_i. \tag{S9}$$

$$= \int \sum_{\forall \bar{l}_{j-1}} \sum_{\forall \bar{y}_{j-1}} \mathbb{E}(Y_j|\bar{H}_{j-1}, \eta) \prod_{k=1}^{j-1} f(Y_k|\bar{H}_{k-1}, \eta)P(L_k = l_k|\bar{H}_{k-1})f(\eta_i)\partial\eta_i. \tag{S10}$$

where η_i denotes the time-invariant unmeasured confounder between treatment regimen and outcome regimen for individual i. Equation (S10) can be noted since $L_k \perp\!\!\!\perp \eta_i$ in our formulation.

C. Derivation of covariate eliminating restrictions

We maximize the score function for treatment regimen A_{ij} using the weights $SW_{ij}^A(\lambda)$ with unknown parameters λ as

$$\prod_{i=1}^{n}\prod_{j=1}^{m_i} \left\{ \frac{\partial}{\partial\alpha} \prod_{k=1}^{j} P_\alpha(A_{ik}|\bar{H}_{ik-1}; \alpha) \right\}^{SW_{ij}^A(\lambda)} \Bigg|_{\alpha_b=\hat{\alpha}, \alpha_d=0} = 0.$$

Since A_{ij} is the binary treatment assignment for i^{th} individual at j^{th} time interval, we represent the score function using the binomial kernel as

$$\sum_{i=1}^{n}\sum_{j=1}^{m_i} SW_{ij}^A(\lambda) \sum_{k=1}^{j} \frac{\partial}{\partial\alpha} \left[A_{ik} \times log(P_\alpha(A_{ik}|\bar{H}_{ik-1})) + (1 - A_{ik}) \times log(1 - P_\alpha(A_{ik}|\bar{H}_{ik-1}))\right]\Big|_{\alpha_b=\hat{\alpha}, \alpha_d=0} = 0.$$

We may further simplify the score function as

$$\Rightarrow \sum_{i=1}^{n}\sum_{j=1}^{m_i} SW_{ij}^{A}(\lambda)\sum_{k=1}^{j}\frac{\partial}{\partial\alpha}\left[log(P_\alpha(1-A_{ik}|\bar{H}_{ik-1})) + A_{ik}\times log\frac{P_\alpha(A_{ik}|\bar{H}_{ik-1})}{(1-P_\alpha(A_{ik}|\bar{H}_{ik-1}))}\right]\Bigg|_{\alpha_b=\hat{\alpha},\alpha_d=0} = 0 \quad \text{(S11)}$$

$$\Rightarrow \sum_{i=1}^{n}\sum_{j=1}^{m_i} SW_{ij}^{A}(\lambda)\sum_{k=1}^{j}\frac{\partial}{\partial\alpha}\left[-log(1+exp(\alpha^T\bar{H}_{ik-1})) + A_{ik}\times\left(\alpha^T\bar{H}_{ik-1}\right)\right]\Bigg|_{\alpha_b=\hat{\alpha},\alpha_d=0} = 0 \quad \text{(S12)}$$

$$\Rightarrow \sum_{i=1}^{n}\sum_{j=1}^{m_i} SW_{ij}^{A}(\lambda)\sum_{k=1}^{j}\left[-\frac{\bar{H}_{ik-1}\times exp(\alpha^T\bar{H}_{ik-1})}{1+exp(\alpha^T\bar{H}_{ik-1})} + A_{ik}\times\bar{H}_{ik-1}\right]\Bigg|_{\alpha_b=\hat{\alpha},\alpha_d=0} = 0 \quad \text{(S13)}$$

$$\Rightarrow \sum_{i=1}^{n}\sum_{j=1}^{m_i} SW_{ij}^{A}(\lambda)\sum_{k=1}^{j}\left[(A_{ik}-\hat{e}_{ik}^{A})\times\bar{H}_{ik-1}\right]\Bigg|_{\alpha_b=\hat{\alpha},\alpha_d=0} = 0 \quad \text{(S14)}$$

where $\hat{e}_{ik}^{A} = P(A_{ik}|\bar{H}_{ik-1})$. In the last equation (S14), we notice that the treatment residuals (i.e. $A_{ik}-\hat{e}_{ik}^{A}$) are orthogonal with respect to covariate history $\bar{H}_{ik-1}$ at each time interval. Without loss of generality, we may also extend the above derivations to irregular visits V_{ij}. We use equation (S14) as the objective function and we solve for the unknown parameters λ.

D. Derivation of time-invariant latent restrictions

The calibrated treatment weights are defined as $SW_{ij}^{A}(\lambda) = SW_{ij}^{A}exp(K\lambda)$. The data-dependent restrictions (in Section B) are incorporated inside the $K \in \mathbb{R}^{N\times r}$ matrix. We solve for the vector of unknown parameters $\lambda \in \mathbb{R}^r$ using these calibrated restrictions. We build the system of simultaneous equations as

$$\sum_{i=1}^{n}\sum_{j=1}^{m_i} SW_{ij}^{A}(\lambda)\sum_{k=1}^{j}\left[(A_{ik}-\hat{e}_{ik}^{A})\times\bar{H}_{ik-1}\right]\Bigg|_{\alpha_b=\hat{\alpha},\alpha_d=0} = 0 \quad \text{(S15)}$$

$$\iff \sum_{i=1}^{n}\sum_{j=1}^{m_i} SW_{ij}^{A}(\lambda)\sum_{k=1}^{j}\left[(A_{ik}-\hat{e}_{ik}^{A})\times\begin{pmatrix}1\\ L_{ik-1}\\ \eta_i\end{pmatrix}\right]\Bigg|_{\alpha_b=\hat{\alpha},\alpha_d=0} = 0 \quad \text{(S16)}$$

We suppress the notation for $|_{\alpha_b=\hat{\alpha},\alpha_d=0}$ for each simultaneous equations below:

$$\iff \begin{cases}\sum_{i=1}^{n}\sum_{j=1}^{m_i} SW_{ij}^{A}(\lambda)\sum_{k=1}^{j}\left[(A_{ik}-\hat{e}_{ik}^{A})\times 1\right] & = 0\\ \sum_{i=1}^{n}\sum_{j=1}^{m_i} SW_{ij}^{A}(\lambda)\sum_{k=1}^{j}\left[(A_{ik}-\hat{e}_{ik}^{A})\times L_{ik-1}\right] & = 0\\ \sum_{i=1}^{n}\sum_{j=1}^{m_i} SW_{ij}^{A}(\lambda)\sum_{k=1}^{j}\left[(A_{ik}-\hat{e}_{ik}^{A})\times \eta_i\right] & = 0\end{cases} \quad \text{(S17)}$$

$$\iff \begin{cases}\sum_{i=1}^{n}\sum_{j=1}^{m_i} SW_{ij}^{A}(\lambda)\sum_{k=1}^{j} A_{ik} & = \sum_{i=1}^{n}\sum_{j=1}^{m_i} SW_{ij}^{A}(\lambda)\sum_{k=1}^{j}\hat{e}_{ik}^{A}\\ \sum_{i=1}^{n}\sum_{j=1}^{m_i} SW_{ij}^{A}(\lambda)\sum_{k=1}^{j} A_{ik}\times L_{ik-1} & = \sum_{i=1}^{n}\sum_{j=1}^{m_i} SW_{ij}^{A}(\lambda)\sum_{k=1}^{j}\hat{e}_{ik}^{A}\times L_{ik-1}\\ \sum_{i=1}^{n}\eta_i\sum_{j=1}^{m_i} SW_{ij}^{A}(\lambda)\sum_{k=1}^{j} A_{ik} & = \sum_{i=1}^{n}\eta_i\sum_{j=1}^{m_i} SW_{ij}^{A}(\lambda)\sum_{k=1}^{j}\hat{e}_{ik}^{A}\end{cases} \quad \text{(S18)}$$

In equation (S18), we notice that the constraint to balance the latent time-invariant confounder η_i may be represented empirically using the sufficiency conditions as

$$\Leftarrow \begin{cases}\sum_{i=1}^{n}\sum_{j=1}^{m_i} SW_{ij}^{A}(\lambda)\sum_{k=1}^{j} A_{ik} & = \sum_{i=1}^{n}\sum_{j=1}^{m_i} SW_{ij}^{A}(\lambda)\sum_{k=1}^{j}\hat{e}_{ik}^{A}\\ \sum_{i=1}^{n}\sum_{j=1}^{m_i} SW_{ij}^{A}(\lambda)\sum_{k=1}^{j} A_{ik}\times L_{ik-1} & = \sum_{i=1}^{n}\sum_{j=1}^{m_i} SW_{ij}^{A}(\lambda)\sum_{k=1}^{j}\hat{e}_{ik}^{A}\times L_{ik-1}\ .\\ \sum_{j=1}^{m_i} SW_{ij}^{A}(\lambda)\sum_{k=1}^{j} A_{ik} & = \sum_{j=1}^{m_i} SW_{ij}^{A}(\lambda)\sum_{k=1}^{j}\hat{e}_{ik}^{A} \qquad \forall i\end{cases} \quad \text{(S19)}$$

In equation (S19), we notice that if the third equation is satisfied for each time interval j then it will also satisfy the first equation aggregated over i individuals. This leads to the generation of n constraints for each individual i (separately) in the data-dependent K matrix. Now we simplify equation (S19) further by removing the first equation as

$$\Leftarrow \begin{cases}\sum_{i=1}^{n}\sum_{j=1}^{m_i} SW_{ij}^{A}(\lambda)\sum_{k=1}^{j} A_{ik}\times L_{ik-1} & = \sum_{i=1}^{n}\sum_{j=1}^{m_i} SW_{ij}^{A}(\lambda)\sum_{k=1}^{j}\hat{e}_{ik}^{A}\times L_{ik-1}\\ \sum_{j=1}^{m_i} SW_{ij}^{A}(\lambda)\sum_{k=1}^{j} A_{ik} & = \sum_{j=1}^{m_i} SW_{ij}^{A}(\lambda)\sum_{k=1}^{j}\hat{e}_{ik}^{A} \qquad \forall i\end{cases} \quad \text{(S20)}$$

$$\equiv \begin{cases}\sum_{i=1}^{n}\sum_{j=1}^{m_i} SW_{ij}^{A}(\lambda)\sum_{k=1}^{j}\left[(A_{ik}-\hat{e}_{ik}^{A})\times L_{ik-1}\right] & = 0\\ \sum_{j=1}^{m_i} SW_{ij}^{A}(\lambda)\sum_{k=1}^{j}\left[(A_{ik}-\hat{e}_{ik}^{A})\right] & = 0 \qquad \forall i\end{cases} \quad \text{(S21)}$$

We express equation (S21) using the coefficients terms as

$$\begin{cases} \sum_{i=1}^{n} \sum_{k=1}^{j} \left[(A_{ik} - \hat{e}_{ik}^{A}) \times L_{ik-1} \right] & = 0 \\ \sum_{k=1}^{j} \left[(A_{ik} - \hat{e}_{ik}^{A}) \right] & = 0 \qquad \forall i \end{cases} \tag{S22}$$

We enforce the empirical constraints in equation (S21) as a cumulative-time sum over discrete time intervals for each individual i (separately) to account for time-invariant latency η_i. The simultaneous equations (S21) combined with unity and orthogonality constraints are embedded inside the data-dependent restrictions in matrix $\boldsymbol{K} \in \mathbb{R}^{N \times r}$. In particular, we further partition the $\boldsymbol{K}$ matrix using the data-dependent constraints with respect to (i) orthogonality between treatment residual and time-dependent covariates ($\boldsymbol{K}_1$); (ii) unity mean restriction at each discretized time-point ($\boldsymbol{K}_2$) and (iii) latency constraints ($\boldsymbol{K}_3$). We describe the $\boldsymbol{K}$ matrix as the horizontal concatenation of three sub-matrices: $\boldsymbol{K} = \begin{bmatrix} \boldsymbol{K}_1 & \boldsymbol{K}_2 & \boldsymbol{K}_3 \end{bmatrix}$. We describe each matrix containing the data-dependent restrictions as

$$\boldsymbol{K}_1 = \begin{bmatrix} 1 & \sum_{i=1} \sum_{k=1} (A_{ik} - \hat{e}_{ik}^{A}) \times L_{ik-1} \\ 1 & \sum_{i=1} \sum_{k=1}^{2} (A_{ik} - \hat{e}_{ik}^{A}) \times L_{ik-1} \\ 1 & \cdot \\ 1 & \cdot \\ 1 & \sum_{i=2} \sum_{k=1} (A_{ik} - \hat{e}_{ik}^{A}) \times L_{ik-1} \\ 1 & \sum_{i=1} \sum_{k=1}^{2} (A_{ik} - \hat{e}_{ik}^{A}) \times L_{ik-1} \\ 1 & \cdot \\ 1 & \cdot \end{bmatrix}_{N \times 2}$$

$$\boldsymbol{K}_2 = \begin{bmatrix} \mathbb{1}(k=1) & \cdot & \cdot & \cdot & \cdot & \cdot \\ \cdot & \mathbb{1}(k=2) & \cdot & \cdot & \cdot & \cdot \\ \cdot & \cdot & \mathbb{1}(k=3) & \cdot & \cdot & \cdot \\ \cdot & \cdot & \cdot & \cdot & \cdot & \cdot \\ \cdot & \cdot & \cdot & \cdot & \cdot & \cdot \end{bmatrix}_{N \times t}$$

$$\boldsymbol{K}_3 = \begin{bmatrix} \sum_{i=1} \sum_{k=1} (A_{ik} - \hat{e}_{ik}^{A}) & 0 & 0 & \cdots \\ \sum_{i=1} \sum_{k=1}^{2} (A_{ik} - \hat{e}_{ik}^{A}) & 0 & 0 & \cdots \\ \cdot & 0 & 0 & \cdots \\ \cdot & 0 & 0 & \cdots \\ 0 & \sum_{i=2} \sum_{k=1} (A_{ik} - \hat{e}_{ik}^{A}) & 0 & \cdots \\ 0 & \sum_{i=2} \sum_{k=1}^{2} (A_{ik} - \hat{e}_{ik}^{A}) & 0 & \cdots \\ 0 & \cdot & 0 & \cdots \\ 0 & \cdot & 0 & \cdots \\ 0 & 0 & \sum_{i=3} \sum_{k=1} (A_{ik} - \hat{e}_{ik}^{A}) & \cdots \\ 0 & 0 & \sum_{i=3} \sum_{k=2} (A_{ik} - \hat{e}_{ik}^{A}) & \cdots \\ 0 & 0 & \cdot & \cdots \\ 0 & 0 & \cdot & \cdots \\ 0 & 0 & 0 & \cdots \end{bmatrix}_{N \times n}$$

We notice that the total number of rows in each matrix correspond to the simulated data size for each replicate (i.e. N is the total number of simulated observations). Furthermore, the total number of columns in $\boldsymbol{K}$ matrix correspond to the dimension of $\boldsymbol{\lambda}^T \in \mathbb{R}^{n+t+2}$ (i.e. $r = n + t + 2$). We notice that restrictions in matrix $\boldsymbol{K}_3$ are encoded separately in each column for individual i as a cumulative sum over longitudinal follow-up using the index m. The total number of columns in $\boldsymbol{K}_2$ matrix denote the total number of t discrete time interval.

Using this setup, we solve for the system of linear equations as

$$\boldsymbol{K^T SW_{ij}^{A}} \circ \boldsymbol{c}(\boldsymbol{L_{ij}}, \boldsymbol{\lambda}) - \boldsymbol{l} = \boldsymbol{0} \tag{S23}$$

where the non-negative function $\boldsymbol{c}(\boldsymbol{L_{ij}}, \boldsymbol{\lambda})$ is specified as $\boldsymbol{exp}(\boldsymbol{K\lambda})$, $\circ$ denotes element-wise product and $\boldsymbol{\lambda}$ denotes the vector of λ to be estimated. We notice that $\boldsymbol{K}^T \in \mathbb{R}^{r \times N}$ and $\boldsymbol{SW_{ij}^{A}}(\lambda) \in \mathbb{R}^{r \times 1}$ generate r simultaneous equations with the constant vector $\boldsymbol{l} \in \mathbb{R}^{r \times 1}$. Since the system of linear equations are convex with respect to the calibration function $\boldsymbol{exp}(\boldsymbol{K\lambda})$, a unique solution can be found using the $r \times r$ Hessian matrix (i.e. $\boldsymbol{K^T K} \circ \boldsymbol{SW_{ij}^{A}} \circ \boldsymbol{exp}(\boldsymbol{K\lambda})$) to estimate $\boldsymbol{\lambda}$. We fixed all parameter values except for $\boldsymbol{\lambda}$ vector, and we solve for the $\boldsymbol{\lambda}$ vector using the df-SANE algorithm in which $\boldsymbol{K} \in \mathbb{R}^{N \times r}$ characterized the data-dependent

constraints. We notice that the empirical constraints in equation (S21) can be represented using a system of linear equations (S23) using $\boldsymbol{K}_1$, $\boldsymbol{K}_2$ and $\boldsymbol{K}_3$ matrix. Without loss of generality, we may express the same set of arguments to generate the empirical equations for the latent confounder η_i using the irregular visits V_{ij}.
